# Filamin-A Increases the Stability and Plasma Membrane Expression of Polycystin-2

**DOI:** 10.1371/journal.pone.0123018

**Published:** 2015-04-10

**Authors:** Qian Wang, Wang Zheng, Zuocheng Wang, JungWoo Yang, Shaimaa Hussein, Jingfeng Tang, Xing-Zhen Chen

**Affiliations:** 1 Membrane Protein Disease Research Group, Department of Physiology, Faculty of Medicine and Dentistry, University of Alberta, Edmonton, Alberta, Canada; 2 Membrane Protein Disease and Cancer Research Center, Hubei University of Technology, Wuhan, China; Emory University, UNITED STATES

## Abstract

Polycystin-2 (PC2), encoded by the PKD2 gene, is mutated in ~15% of autosomal dominant polycystic kidney disease. Filamins are actin-binding proteins implicated in scaffolding and membrane stabilization. Here we studied the effects of filamin on PC2 stability using filamin-deficient human melanoma M2, filamin-A (FLNA)-replete A7, HEK293 and IMCD cells together with FLNA siRNA/shRNA knockdown (KD). We found that the presence of FLNA is associated with higher total and plasma membrane PC2 protein expression. Western blotting analysis in combination with FLNA KD showed that FLNA in A7 cells represses PC2 degradation, prolonging the half-life from 2.3 to 4.4 hours. By co-immunoprecipitation and Far Western blotting we found that the FLNA C-terminus (FLNAC) reduces the FLNA-PC2 binding and PC2 expression, presumably through competing with FLNA for binding PC2. We further found that FLNA mediates PC2 binding with actin through forming complex PC2-FLNA-actin. FLNAC acted as a blocking peptide and disrupted the link of PC2 with actin through disrupting the PC2-FLNA-actin complex. Finally, we demonstrated that the physical interaction of PC2-FLNA is Ca-dependent. Taken together, our current study indicates that FLNA anchors PC2 to the actin cytoskeleton through complex PC2-FLNA-actin to reduce degradation and increase stability, and possibly regulate PC2 function in a Ca-dependent manner.

## Introduction

Polycystin-2 (PC2 or TRPP2) belongs to the transient receptor potential polycystin (TRPP) subfamily of TRP channels. PC2 is a 968 amino-acid (aa) integral membrane protein with six transmembrane domains and intracellularly localized N- and C-termini. Encoded by the PKD2 gene, PC2 is a Ca-permeable cation channel [1] mainly located on the endoplasmic reticulum (ER) membrane [2], but is also present in the primary cilium [3,4] and plasma membrane (PM) [5]. PC2 membrane targeting is regulated by several factors, such as PC1, phosphofurin acidic cluster sorting proteins and intracellular calcium release [6–9]. Mutations in PKD2 account for 10–15% of the autosomal dominant polycystic kidney disease (ADPKD) [1], a prominent inherited disorder that affects 12.5 million people worldwide. ADPKD is characterized by formation of cysts in kidneys, and to a less extent in liver and pancreas. At the cellular level, ADPKD is associated with elevated cell proliferation, apoptosis, and de-differentiation [10–12]. Until now, the underlying mechanisms of cyst formation have remained ill-defined and no effective therapy has been developed. Previously, we found that PC2 down-regulates cell proliferation via promoting PERK-dependent phosphorylation of eukaryotic initiation factor eIF2α [13]. Mice with either loss- or gain-of-function of PC2 are cystogenic [14,15]. Therefore, it seems that PC2 cellular level has to be regulated within a narrow range.

Filamin-A (FLNA), the first non-muscle actin filament cross-linking protein, was identified in 1975 [16]. The filamin protein family comprises three members, FLNA, filamin-B and-C (FLNC) which share 60–80% sequence homology, of which FLNA is the most abundant and widely distributed [17]. Filamins contain a spectrin-related domain in the N-terminus that directly binds actin and is followed by 24 β-sheet repeats [17,18]. The most C-terminal repeat #24 mediates formation of a homodimer of flexible V-shaped structure that acts as ‘a molecular leaf spring’ to facilitate cross-linking of actin filaments [19]. By cross-linking cortical actin, filamins give cells a dynamic three-dimensional structure. They also interact with a large number of proteins of great functional diversity, indicating that they are versatile scaffolding proteins. Over 90 filamin-interacting partners have so far been identified, including channels, receptors, intracellular signaling molecules, and even transcription factors [20]. Recently, we reported that filamins interact with the epithelial sodium channel (ENaC) on the surface membrane for both structural purposes and functional regulation [21]. The presence of this extensive array of associated proteins may account in part for the fact that mutations in human filamin genes result in a wide range of cell and tissue anomalies, including bone anomalies, periventricular heterotopias, aortic dissection and aneurysm [22–24].

Connections between PC2 and cytoskeleton proteins have been established by several studies. Among PC2-interacting partners identified so far, half are cytoskeleton or cytoskeleton-associated proteins [25]. In 2005, we found that α-actinin, an actin-binding protein important in cytoskeleton organization, cell adhesion, proliferation and migration, interacts with both the intracellular N- and C-termini of PC2 and substantially stimulated its channel function [26]. PC2 channel function is modulated by dynamic changes in actin filament organization in the apical membrane of human syncytiotrophoblast, which is the most apical epithelial barrier that covers the villous tree of human placenta [27]. Further studies suggested that cytoskeleton proteins are likely to mediate the regulation of PC2 channel function by physical forces such as hydrostatic and osmotic pressure in human syncytiotrophoblast [28]. In mitotic spindles of dividing cells PC2 interacts and co-localizes with mDia1, a member of the RhoA GTPase-binding formin homology protein family that participates in cytoskeletal organization [9]. Recently, we demonstrated that C-terminus of filamins directly bind to both the intracellular N- and C- termini of PC2, and that FLNA substantially inhibits PC2 channel activity in a lipid bilayer reconstitution system [21].

In the present study, we explored the role of FLNA in regulating PC2 stability and degradation, using mammalian cultured cells in combination with ^35^S pulse labeling, Western blotting (WB), co-immunoprecipitation (co-IP), cell surface biotinylation, and gene over-expression/knockdown (KD). We also examined the Ca-dependence of the PC2-FLNA binding.

## Materials and Methods

### Antibodies

PC2 (H-280), GFP (B-2), FLNA (E-3), FLNA (H-300), β-actin (C4), Na/K ATPase (H-300) and HSP60 (H-1) antibodies were purchased from Santa Cruz Biotech (Santa Cruz, Dallas, TX, USA). PC2 (1A11) mouse polyclonal antibody [26,29] and PC2 (H-280) was used to detect native PC2. Antibody E-3 against the N-terminus of FLNA was used to detect full-length FLNA while antibody H-300 was used to detect both the full length and C-terminus of FLNA (FLNAC, aa 2150–2647). Goat anti-GFP (EU4) was purchased from Eusera (Eusera, Edmonton, AB, Canada). Secondary antibodies were purchased from GE Healthcare (Baie d’Urfe, QC, Canada).

### Cell lines and transfection

M2 and A7 PC2 stable cell lines, as previously described [21], were maintained with hygromycin (100 μg/ml) (M2) or hygromycin plus G418 (300 μg/ml) (A7) (Invitrogen Canada, Burlington, ON, Canada). Full length human FLNA plasmid pcDNA3-myc-FLNA (ID 8982) was purchased from Addgene (Addgene, Cambridge, MA, USA). FLNA shRNA and the corresponding empty vector p/PUR/U6 as a negative control were generous gifts of Dr. Z. Shen (Cancer Institute of New Jersey, New Brunswick, NJ, USA). Briefly, HeLa and A7 cells were transfected with either the empty vector or FLNA shRNA using the Lipofectamine 2000 reagent (Invitrogen Canada) according to the manufacturer’s instructions. After 48 hours (hr) of transfection, 5 μg/ml of puromycin (Invitrogen Canada) was added to the medium for selection of FLNA stable KD cells. FLNA siRNA pairs 5601 (5’-CCCAUGGAGUAGUGAACAATT-3’ and 5’-UUGUUCACUACUCCAUGGGTG-3’), 7116 (5’-CAGAAAUUGACCAAGAUAATT-3’ and 5’-UUAUCUUGGUCAAUUUCUGTG-3’), and 371 (5’-GGAAGAAGAUCCAGCAGAATT-3’ and 5’UUCUGCUGGAUCUUCUUCCAC-3’), were ordered from GenePharma (GenePharma, Shanghai, China). FLNA siRNA, or control siRNA were transfected into HeLa, human embryonic kidney (HEK293) and mouse inner medullary collecting duct (IMCD) cells using HiPerFect Reagent (Qiagen, Hilden, Germany) according to the manufacturer’s instructions (siRNA final concentration: 20 nM). Cells were transfected for a second time after 24 hr. The efficiency of shRNA and siRNA KD was assessed by immunoblotting (IB). Transient transfection with previously described pEGFPC2 plasmids (with GFP tag) harboring PC2, the C-terminus of PC2 (PC2C, aa 682–968), the N-terminus of PC2 (PC2N, aa 1–215) or His-FLNAC [21] was also performed using Lipofectamine 2000 and the expression was assessed 48 hr after transfection. For IMCD and M2 cells, a second transfection was performed 24 hr after the first transfection to increase transfection efficiency. Cells were harvested 72 hr after the first transfection.

### ^35^S pulse labeling

^35^S pulse labeling assay was carried out to study the effect of FLNA on PC2 synthesis. Equal number of M2 and A7 cells either stably or transiently expressing GFP-tagged PC2 were plated in 60-mm dishes for 1 hr starvation in the pre-labeling medium L-methionine and L-cysteine free Dulbecco's modified Eagle's medium (DMEM) with 10% fetal bovine serum (FBS) and penicillin/streptomycin (Invitrogen Canada). This was followed by pulse-labeling with 250 μCi of [^35^S]methionine/cysteine EXPRE^35^S Protein Labeling Mix (PerkinElmer, Woodbridge, ON, Canada) for 30 minutes (min). Cells were harvested in ice-cold CelLytic-M lysis buffer (Sigma-Aldrich Canada, Oakville, ON, Canada) supplemented with protease inhibitor cocktail (Sigma-Aldrich Canada and 500 μg total protein was used for immunoprecipitation (IP) with anti-GFP (EU4) and magnetic Dynabeads (Invitrogen Canada). The resulting precipitates were subject to SDS-PAGE and autoradiography.

### Cell surface biotinylation

Cells were grown to 90% confluency in 100-mm dishes, washed with ice-cold PBS and borate buffer (10 mM boric acid, 154 mM NaCl, 7.2 mM KCl, 1.8 mM CaCl_2_, pH 9.0), and then incubated with 0.5 mg/ml Pierce EZ-LinkTM Sulfo-NHS-SS-Biotin (Fisher Scientific Canada, Toronto, ON, Canada) at 4°C with agitation for 30 min. After washing with quenching buffer (192 mM glycine, 25 mM Tris, pH 8.3), cells were lysed in ice-cold CelLytic-M reagent supplemented with protease inhibitor cocktail to make 2–5 μg/μl total protein lysate. The biotinylated and flow-through intracellular proteins were separated using 50 μl of Pierce Avidin Agarose (Fisher Scientific Canada, Markham, ON, Canada) by incubation overnight at 4°C and subsequent centrifugation. After intensive washing in the NP40 buffer (50 mM Tris pH 7.5, 150 mM NaCl, 1% NP40) with protease inhibitor cocktail, biotinylated proteins were resuspended in the SDS sample buffer and eluted from beads by heating at 65°C for 5 min. An equal amount of biotinylated proteins and 20 μg of total and intracellular proteins were separated on 8% SDS-PAGE for IB.

### Degradation analysis

Degradation assays were based on the use of protein synthesis inhibitor cycloheximide (CHX) and proteasome inhibitor MG132 (Sigma-Aldrich Canada). Equal number of cells were plated into 6-well plates, treated with CHX (50 μM in DMSO) or MG132 (10μM in DMSO). DMSO was used as control. For CHX treatment, cells were lysed at different time points (0, 2, 4 and 8 hr). For MG132 treatment, cells were lysed at the same time points. Cells were harvested into samples with equal volume (ie equal cell number). The lysates were loaded for WB and signal density was measured by ImageJ (National Institute of Health, Bethesda, MD, USA).

### Co-immunoprecipitation (co-IP)

Cells growing on 100-mm dish were harvested in 1 ml ice-cold CelLytic-M lysis buffer according to the manufacturer’s instructions. 500 μg total proteins from postnuclear supernatant were incubated with 2 μg primary antibodies at 4°C for 4 hr, followed by overnight incubation with gentle shaking upon the addition of 50 μl protein G sepharose (Thermo Fisher Scientific, Rockford, IL, USA). The precipitates absorbed to protein G sepharose were resuspended in SDS sample buffer and subjected to SDS-PAGE, followed by IB. For Ca dependent co-IP experiments, 1 mM Ca and 1 mM EGTA were added to the lysis and washing buffers.

### Far Western blotting (WB)

Far WB was performed as previously described [30]. Briefly, 50 μg protein lysates from M2 and A7 cells stably expressing PC2 were separated by SDS-PAGE. After transfer onto nitrocellulose membranes, proteins were denatured, renatured and then incubated with *E*.*coli* purfied GST-PC2C with or without His-FLNAC, as previously used [21]. After washing, proteins were detected with FLNA and PC2 antibody. FLNA-deficient M2 cells served as a negative control.

### Data analysis

IB signals were quantified by ImageJ and data were analyzed and plotted using SigmaPlot 12 (Systat Software, San Jose, CA, USA), and expressed as mean ± SEM (N), where SEM represents the standard error of the mean and N indicates the number of experimental repeats. Paired or unpaired t-test was used to compare two sets of data. Probability values (p) of less than 0.05 and 0.01 were considered significant (*) and very significant (**), respectively.

## Results

### Effect of FLNA on the steady-state level of PC2

**Fig 1:**
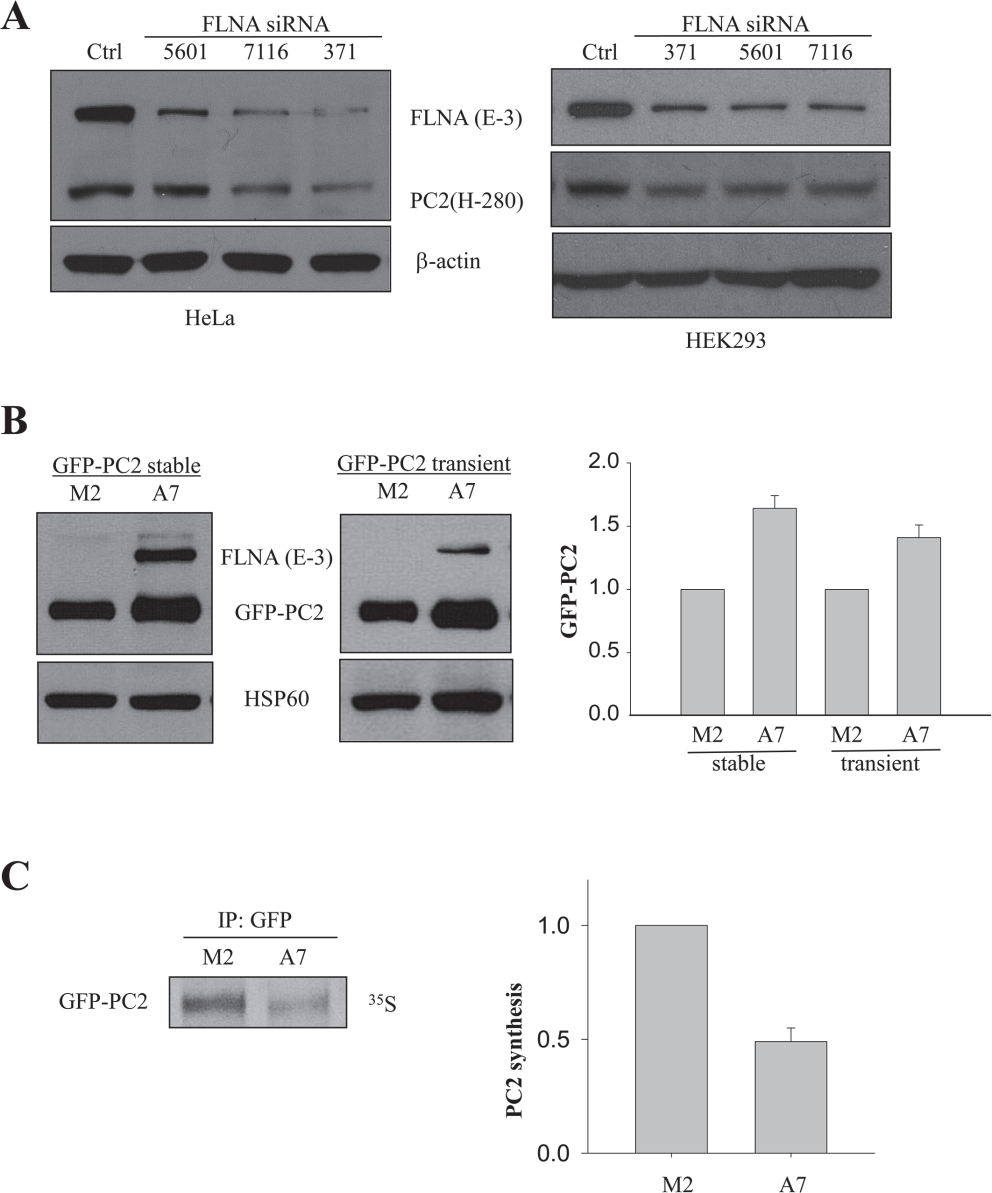
Effects of FLNA on PC2 protein expression and PC2 synthesis. A, WB showing endogenous PC2 level in HeLa (*Left panel*) and HEK293 (*Right panel*) cells with FLNA KD by siRNA 5601, 7116 and 371, respectively. The numbers indicate the nucleotide positions in the FLNA mRNA open reading frame where the siRNA sequence starts. β-actin was used as a loading control. B, *left panel*, data from WB showing the expression of PC2 in M2 and A7 cells stably expressing GFP-PC2. GFP (B-2) antibody was used to detect GFP-PC2. *Middle panel*, PC2 expression in M2 and A7 cells transiently expressing GFP-PC2. *Right panel*, comparison between averaged GFP-PC2 levels normalized by HSP60 in M2 and A7 cells under stable and transient expression conditions. C, *left panel*, representative data from M2 and A7 cells showing GFP-PC2 synthesis assessed by ^35^S pulse labeling. Anti-GFP (EU4) was used to precipitate GFP-PC2. *Right panel*, comparison between averaged GFP-PC2 syntheses in M2 and A7 cells (N = 4; p = 0.004).

We recently reported that FLNA reduces PC2 channel activity through direct binding with both the N- and C-termini of PC2 [21]. Here we found by WB analysis that in HeLa and HEK293 cells the endogenous PC2 expression level substantially decreases in the presence of FLNA knockdown (KD) by three different siRNA pairs (Fig 1A). These data show a clear association between the PC2 and FLNA levels. A similar effect of FLNA KD was also observed in IMCD cells (data not shown). The use of the three effective FLNA siRNA pairs strongly indicates a specific effect of FLNA KD on PC2 expression. We next used human melanoma M2 cells deficient of filamins and FLNA-replete A7 cells for similar experiments. We found that the steady-state level of stably and transiently expressed GFP-PC2 in A7 cells is 64 ± 10% (N = 5; p < 0.001) and 41 ± 10% (N = 3; p < 0.001), respectively, more than that in M2 cells (Fig 1B). These data let us think that the presence of FLNA may lead to higher PC2 protein synthesis or lower PC2 protein degradation. For this we performed ^35^S pulse labeling in combination of co-IP with GFP antibody and magnetic Dynabeads to measure the synthesis of GFP-PC2. WB analysis did not reveal an increased GFP-PC2 protein synthesis (for 30 min) in FLNA-containing A7 cells compared with M2 cells (Fig 1C), suggesting that PC2 in A7 cells may have a lower degradation rate than in M2 cells. These data together let us reason that FLNA may have inhibited cellular PC2 degradation.

### Effect of FLNA on PC2 degradation

**Fig 2:**
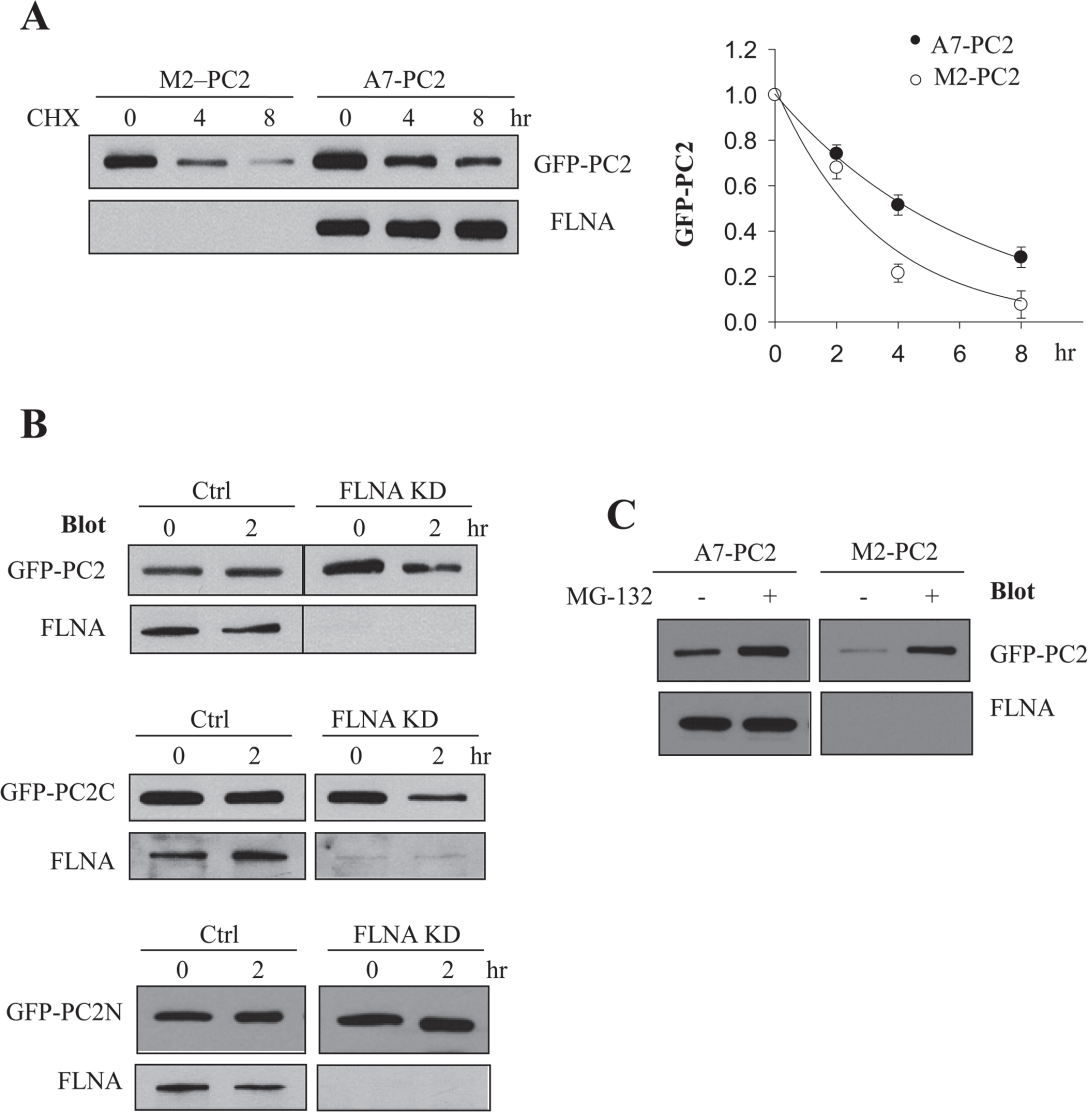
Effect of FLNA on PC2 degradation. A, *left panel*, representative data from WB showing PC2degradation in M2 and A7 cells using cycloheximide (CHX) to block protein synthesis for various time (in hr). *Right panel*, data on PC2 degradation from 4 independent experiments were normalized, averaged and fitted with exponential decay curves Y(t) = 2^-t/τ^, where τ represents the protein half-life. B, representative data showing degradation of GFP-PC2 (*upper panel)*, GFP-PC2C (*middle panel*) and GFP-PC2N (*lower panel*) transiently expressed in A7 cells with FLNA KD or control (Ctrl). C, representative data showing proteasome-dependent inhibition of degradation of GFP-PC2 stably expressed in A7 and M2 cells by MG-132. Cells were treated with MG-132(10 μM) or DMSO (negative control) for 4 hr before proceeding to WB with FLNA and PKD2 antibodies.

We next examined the effect of FLNA on degradation of GFP-PC2 in A7 and M2 stable cell lines. For this, we used cycloheximide (CHX) to stop the biosynthesis and chased the PC2 expression at different time points by WB. We found that PC2 degradation is significantly slower in FLNA-containing A7 cells than in M2 cells (Fig 2A). In average, the half-life of PC2 protein in A7 cells was 4.4 ± 0.2 hr (N = 4) while that in M2 cells was only 2.3 ± 0.5 hr (N = 4). Similar results were obtained for transiently expressed GFP-PC2 in A7 cells with or without FLNA KD, ie, FLNA KD in A7 cells speeded up PC2 degradation (Fig 2B, *upper panel*). Interestingly, although both PC2C and PC2N bind filamins, we found that FLNA only reduces the degradation of PC2C (Fig 2B, *middle panel*), but not PC2N (Fig 2B, *lower panel*) in A7 cells. Our data together indicated that FLNA represses PC2 degradation through interaction with its C-terminus. We and other researchers previously found that PC2 undergoes proteasome degradation [31,32]. To determine whether FLNA affects the pathway through which PC2 is degraded, we utilized proteasome inhibitor MG-132 and found that incubation with MG-132 for 4 hr substantially blocks PC2 degradation in both A7 and M2 cells (Fig 2C), demonstrating that PC2 degradation is still proteasome dependent in the absence of filamin.

### Effect of FLNA on the plasma membrane (PM) PC2 expression

**Fig 3:**
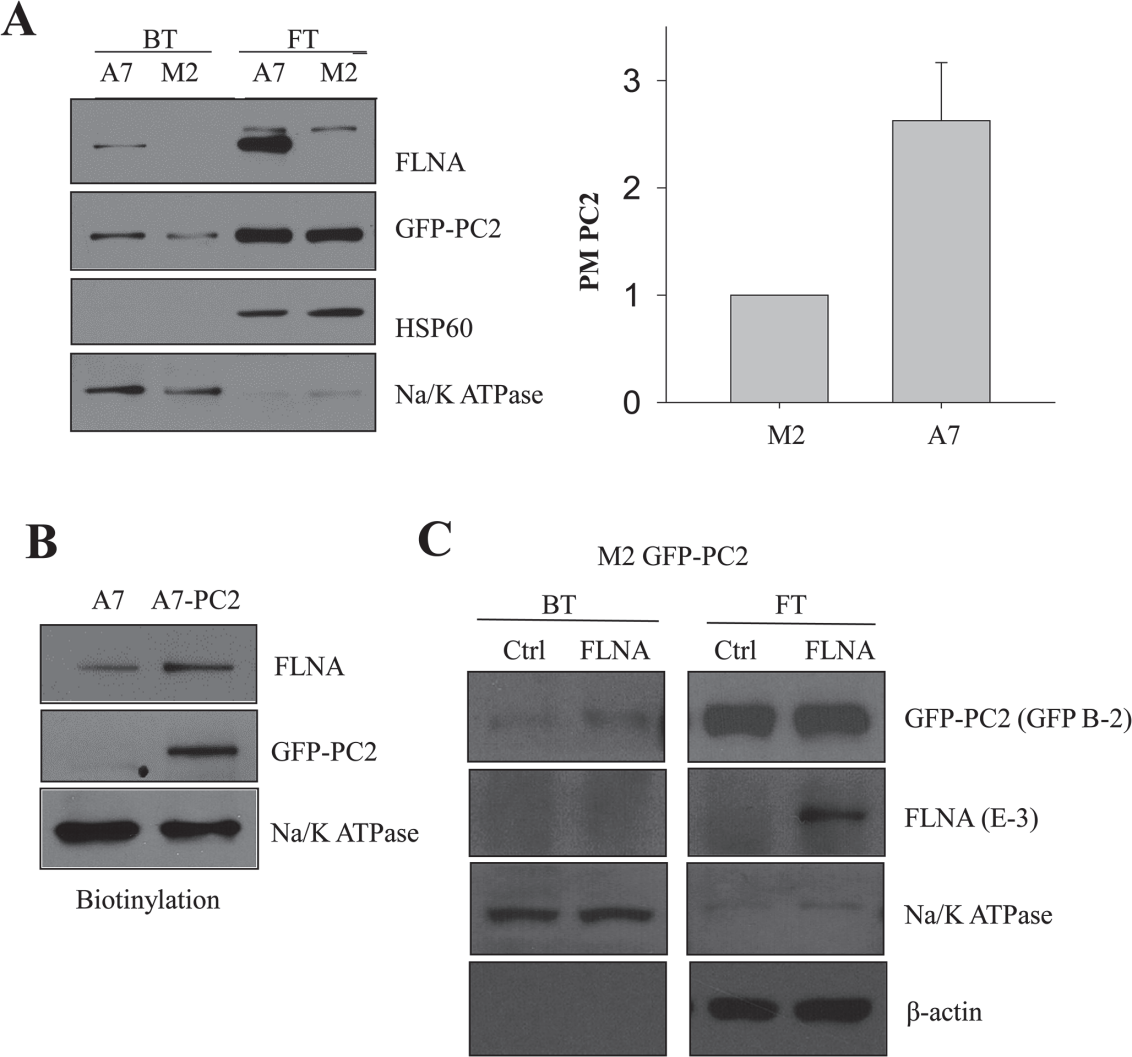
Effect of FLNA on PC2 PM expression. A, *left panel*, cell surface biotinylation was performed to assess GFP-PC2 PM expression in A7 and M2 cells. Shown are data from biotinylated (BT) and flow-through (FT) lysates. *Right panel*, comparison between averaged GFP-PC2 surface membrane expression normalized by Na/K ATPase in M2 and A7 cells (N = 3; p = 0.01). B, biotinylation assays using native A7 (Ctrl) and A7 PC2 stable cells to determine the effect of surface PC2 on the recruitment of FLNA to the PM. C, biotinylation assays using M2 cells with and without transient expression of wild type FLNA transient expression to determine the rescue effect of FLNA on M2 cells.

By use of surface biotinylation, we found that more PC2 is present on the PM of A7 cells compared to M2 cells (Fig 3A). In average, surface PC2 in A7 cells increased by 1.6 ± 0.5 fold (N = 3, p = 0.01) of that in M2 cells. Because the total PC2 expression in A7 cells was only increased by 0.6 fold of that in M2 cells (Fig 1A), this result suggests that FLNA has a stronger stabilizing effect on PM PC2 than intracellular PC2. Interestingly, we detected much more FLNA in the biotinylated A7 cell lysate with PC2 over-expression than in control lysates (Fig 3B), indicating that more FLNA molecules bound with PM PC2 were pulled down under this condition. In fact, we recently reported a similar phenomenon for the scaffolding protein, receptor for activated C kinase RACK1, that interacts with surface membrane TRPP3 channel, a homolog of PC2 [33]. We next examined whether M2 cells transiently transfected with wild type FLNA mimics A7 cells in terms of regulating PM PC2. By WB and biotinylation assays we found that PM PC2 was increased in the presence of FLNA while the flow-through PC2 remained unaffected (Fig 3C). We noticed that FLNA over-expression is rather modest, possibly due to its large size, but still results in an increased PM PC2.

### Effects of FLNAC on the FLNA-PC2 interaction and PC2 expression

**Fig 4:**
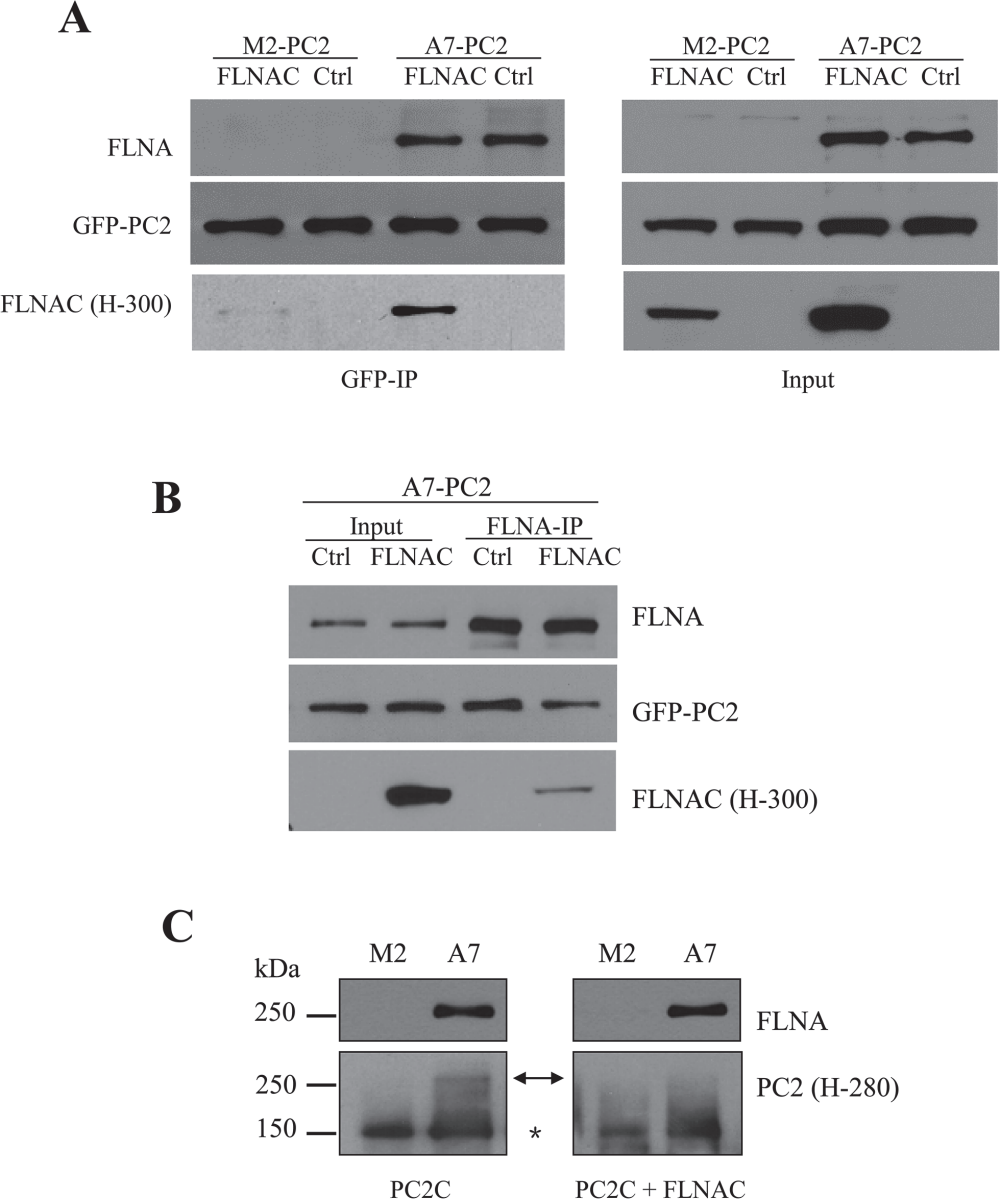
Effect of FLNAC on the FLNA-PC2 interaction. All data are from three or more independent experiments. A, representative data from co-IP showing interaction of GFP-PC2 with FLNA and FLNAC in M2 and A7 cells. B, representative data showing the effect of blocking peptide FLNAC on the FLNA-PC2 interaction in A7 cells. C, Far WB showing the competition of FLNAC with PC2C for binding to FLNA. Lysates of M2 and A7 cells (stably expressing GFP-PC2) were separated by SDS-PAGE and transferred to nitrocellulose membrane. Proteins were denatured, renatured and then incubated with purified GST-PC2C and none (*left panel*) or His-FLNAC (*right panel*). Bound protein was detected by PC2 (H-280) antibody. The arrow (←) indicates PC2C signal detected at the site of FLNA. The star (*) indicates stably expressed GFP-PC2 signal, as a control.

Because PC2 binds FLNA through domain FLNAC (aa 2150–2647) [21], we next utilized FLNAC as a potential blocking peptide to explore how FLNA regulates surface membrane expression of PC2. For this we transiently transfected FLNAC into M2 and A7 cells stably expressing GFP-PC2 and performed co-IP with GFP antibody. The interaction of FLNAC with PC2 was detected in M2 and A7 cells, as expected (Fig 4A). Over-expression of FLNAC reduces the PC2-FLNA interaction in A7 cells (Fig 4B), indicating that FLNAC effectively acts as a blocking peptide. Interestingly, we also observed an interaction between FLNA and FLNAC (Fig 4B), consistent with the fact that FLNA molecules form dimers through their C-terminus [19], which together suggests the presence of complex nPC2-FLNA-FLNAC, where n indicates the number of PC2 molecules in the complex. To further document the role of FLNAC as a blocking peptide in the PC2-FLNA interaction, we performed Far WB assays using A7 and M2 cells. PC2C signal was detected on the membrane at the position/size of FLNA in A7, but not in M2 cell lysate after incubating with GST-PC2C protein purified from *E*. *coli* (Fig 4C, *left panel*), indicating direct binding between PC2C and FLNA. Then, co-incubation of purified His-FLNAC (from *E*. *coli* as well) and purified GST-PC2C reduced the PC2C signal (Fig 4C, *right panel*), indicating that FLNAC competed with PC2C for binding with FLNA.

**Fig 5:**
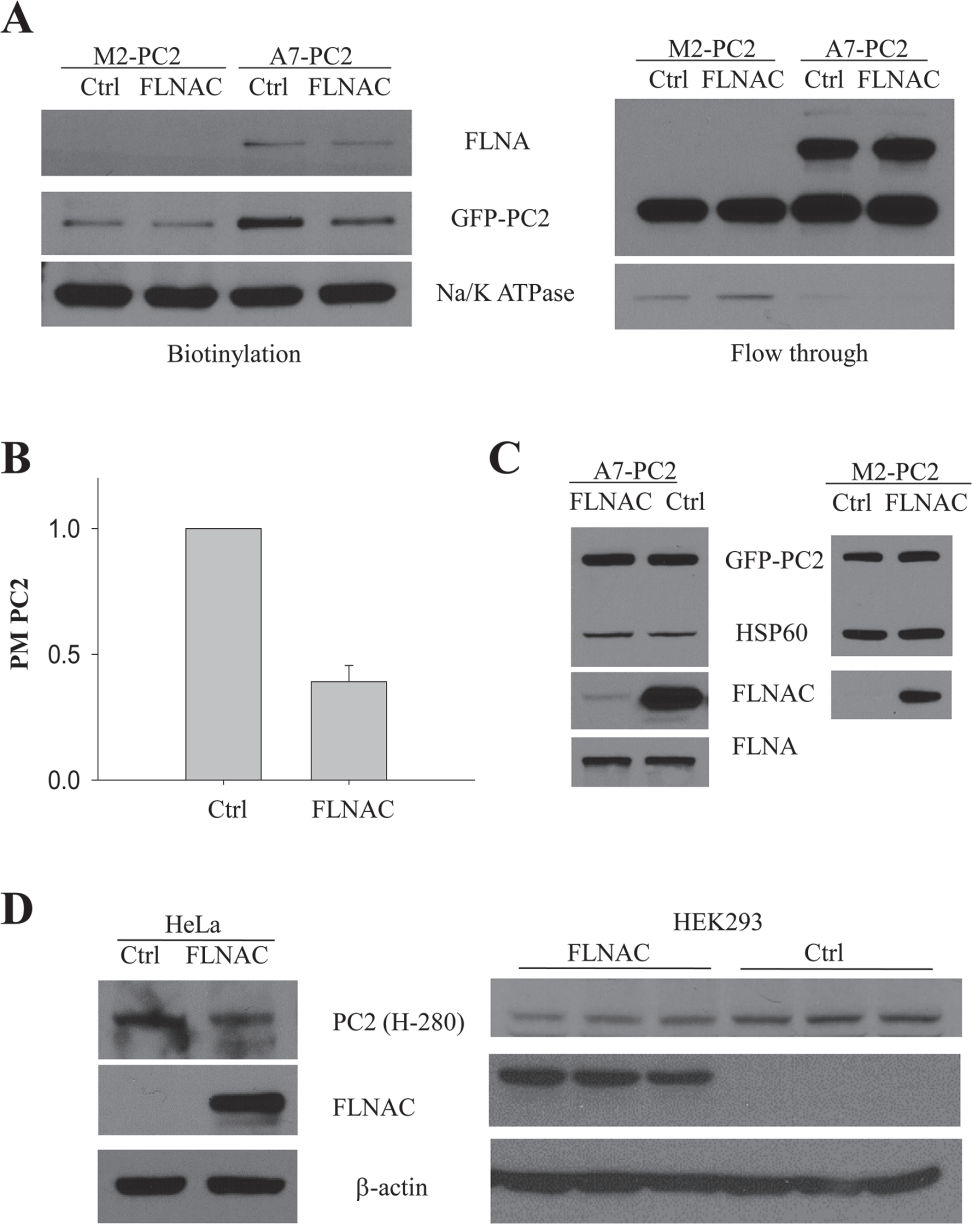
Effect of FLNAC on the surface and total PC2 expression. A, representative biotinylation data showing the effect of FLNAC on the PM (biotinylation) and intracellular (FT) GFP-PC2 expression in M2 and A7 cells. B, averaged PC2 PM expression in A7 cells showing the effect of FLNAC over-expression. FLNAC reduced PC2 PM expression to 46% ± 12% (N = 3; p < 0.001) of the control level. C, representative data showing the effect of FLNAC on the total PC2 expression in GFP-PC2 stably expressing A7 (*left panel*) (N = 3; p = 0.01) and M2 cells (*right panel*) (N = 3; p = 0.16). HSP60 was used as a loading control. D, WB data showing the effect of FLNAC on the endogenous PC2 level in HeLa (*left panel*), HEK293 (*middle panel*) and IMCD (*right pane*l) cells detect by antibody H-280. β-actin was used as a loading control (N = 3).

We reasoned that if the binding domain of FLNA (FLNAC) is sufficient to prevent PC2 degradation, FLNAC over-expression would prolong PC2 half life in M2 cells. On the other hand, if full-length FLNA is required to maintain the stability of PC2, then through disrupting the PC2-FLNA interaction FLNAC should destabilize PC2 in A7 cells but not in M2 cells. To test this hypothesis, we over-expressed FLNAC in A7 and M2 cells and measured the steady-state level of the PM and total PC2. FLNAC significantly reduced the PM level of PC2 (Fig 5A and 5B) in A7 cells while it had no or a very modest effect on the flow-through or total PC2 (Fig 5A, *right panel*, and 5C, *left panel*), and indeed had no effect on either the PM or total PC2 in M2 cells (Fig 5A and 5C, *right panel*). Further, over-expression of FLNAC in HeLa, HEK293 and IMCD cells also resulted in a decreased endogenous PC2 level (Fig 5D). These data together indicated that while full-length FLNA maintains the stability of PC2, FLNAC decreases PC2 stability through interfering with the FLNA-PC2 binding.

### Role of the PC2-FLNA-actin complex in surface PC2 stabilization

**Fig 6:**
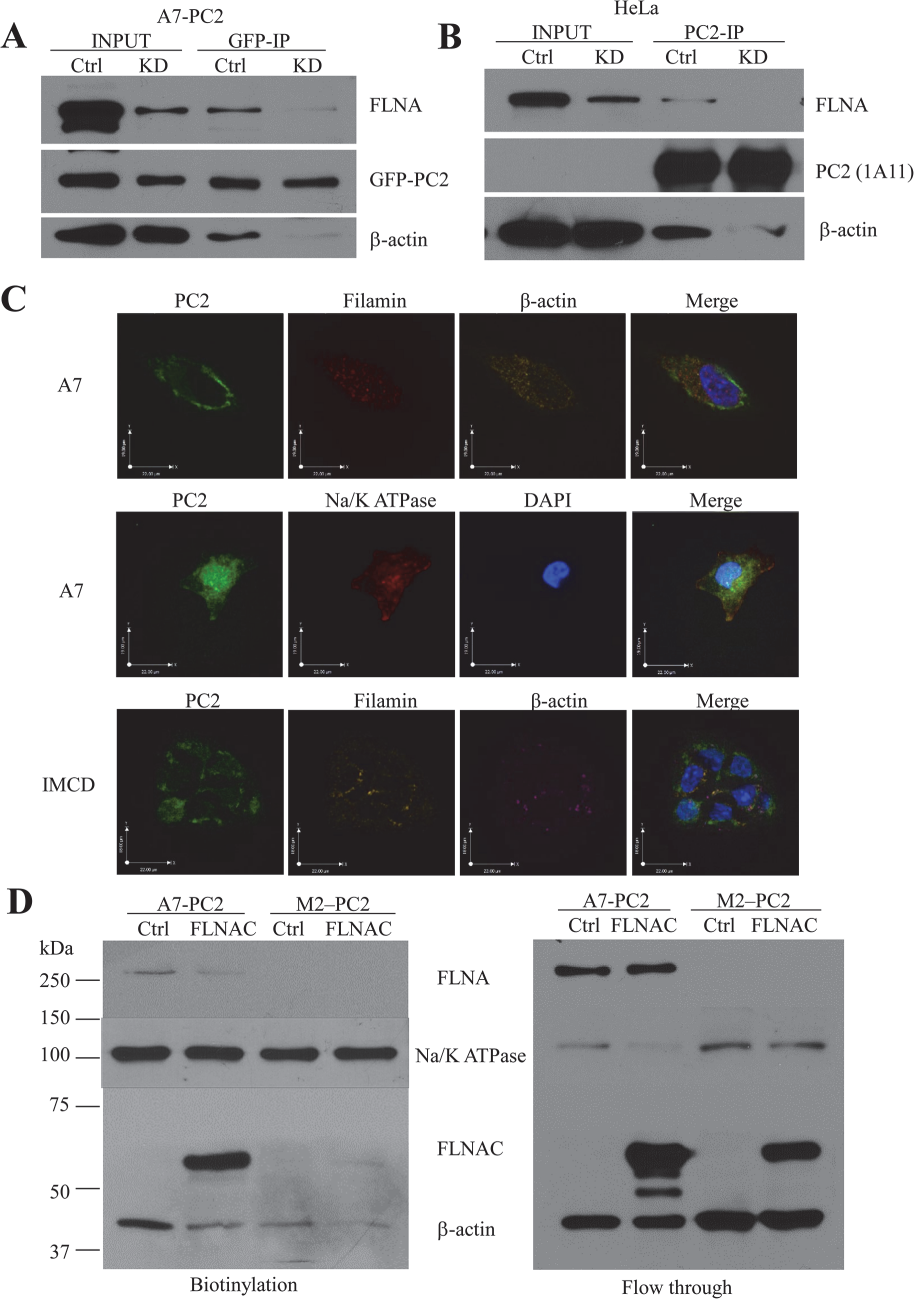
Role of FLNA in, and effect of FLNAC on, the interaction of PC2 with actin. A, effect of FLNA KD on the PC2-actin interaction revealed by co-IP with anti-GFP (EU4) antibody in A7 PC2 stable cells. B, effect of FLNA KD on the PC2-actin interaction revealed by co-IP with PC2 (H-280) antibody in HeLa cells. C, localization of PC2, filamin-A and actin in A7 (*upper panel*) and IMCD *(lower panel)* cells, and co-localization of PC2 and Na/K ATPase in A7 cells were determined by immunofluorescence assays, as previously described [21]. GFP-PC2 are stably expressed in A7 and IMCD cell lines. Primary antibodies against FLNA (H-300), β-actin (C-4), and Na/K ATPase (H-300) were used. Rabbit Cy3- and mouse Cy5-conjugated secondary antibodies were used to detect FLNA (or Na/K ATPase) and actin, respectively. Images were acquired using AIVI spinning disc confocal microscopy with x60 objective. D, effect of FLNAC on the FLNA-mediated PC2-actin interaction using A7 and M2 (as control, no FLNA) cells revealed by biotinylation assays that showed recruitment of actin to the PM.

Since FLNAC decreased the interaction of PC2 with full-length FLNA and seemed to have destabilized surface PC2 in A7 cells, and FLNA is a well known actin-binding protein, we reasoned that FLNA stabilizes PC2 on the cell surface through anchoring to actin filaments. To test this, we first performed co-IP assays to prove the presence of the PC2-FLNA-actin complex. We utilized A7 GFP-PC2 stable cells, with either control (Ctrl) or FLNA KD. We found that both FLNA and β-actin were precipitated by GFP antibody (Fig 6A). FLNA KD dramatically decreased the binding strength of β-actin with PC2, indicating that FLNA is an important mediator for the PC2-β-actin interaction. Furthermore, similar results were obtained using endogenous PC2 in HeLa cells with or without FLNA KD, i.e., FLNA KD much reduced the interaction strength between endogenous PC2 and β-actin (Fig 6B). Given the fact that FLNA binds directly with actin [34], our data together strongly show that PC2, FLNA and β-actin are in the same protein complex, presumably in the form of PC2-FLNA-actin. We also performed immunofluorescence assays with A7 and IMCD cells over-expressing GFP-PC2 to show the relative localization of PC2, FLNA and actin (Fig 6C, *upper and lower panels*). The PM expression of PC2 in A7 cells was also demonstrated by its colocalization with Na/K ATPase (Fig 6C, *middle panel*).

Next, we performed biotinylation assay to check the PC2-FLNA-β-actin complex on the cell surface. We found that in A7 cells, a larger proportion of PC2 is expressed on the cell surface compared to M2 cells (Fig 5A). And correspondingly, more β-actin was detected in the biotinylation lysate of A7 cells than M2 cells (Fig 6D), presumably due to the presence of FLNA in A7 cells. However, in M2 cells, PC2 was also able to bind β-actin at a lower strength, possibly through other interacting partners (Fig 6D). These data suggest that FLNA tethers the PM PC2 to the actin network to prevent rapid internalization as seen in M2 cells. Interestingly, FLNAC over-expression in A7 cells substantially reduced the PM localized PC2- FLNA- β-actin complex (Figs 5A and 6D), presumably because the competition between FLNAC and FLNA for binding PC2 resulted in destabilization of the complex. Thus, because FLNAC does not bind with actin [34], our data indicate that, by reducing the PC2-FLNA interaction, FLNAC prevents PC2 from anchoring to the actin filament, thereby destabilizing PM PC2 and decreasing its PM expression. In summary, these data demonstrate that FLNA stabilizes PC2 on the PM by forming PC2-FLNA-actin complex to anchor it to the actin filament.

The stabilizing effect of actin may also contribute to a higher amount of FLNAC detected in the PM PC2 precipitate in A7 cells than in M2 cells (Fig 6D). As filamin mainly exists as dimers in cells, complex nPC2-FLNAC-FLNAC would be present in M2 cells. However, as this complex is unable to bind to actin due to the absence of the actin-binding domain localized on the FLNA N-terminus, it should be less stable than complex nPC2-FLNA-FLNAC in A7 cells that is able to bind to actin. Further, the fact that both the PM PC2 and total FLNAC levels in A7 cells are much higher than those in M2 cells (Figs 3A and 4A) should be another contributing factor for a higher amount of FLNAC bound to the PM PC2 in A7 cells.

### Roles of Ca on the physical interaction of PC2 and FLNA

**Fig 7:**
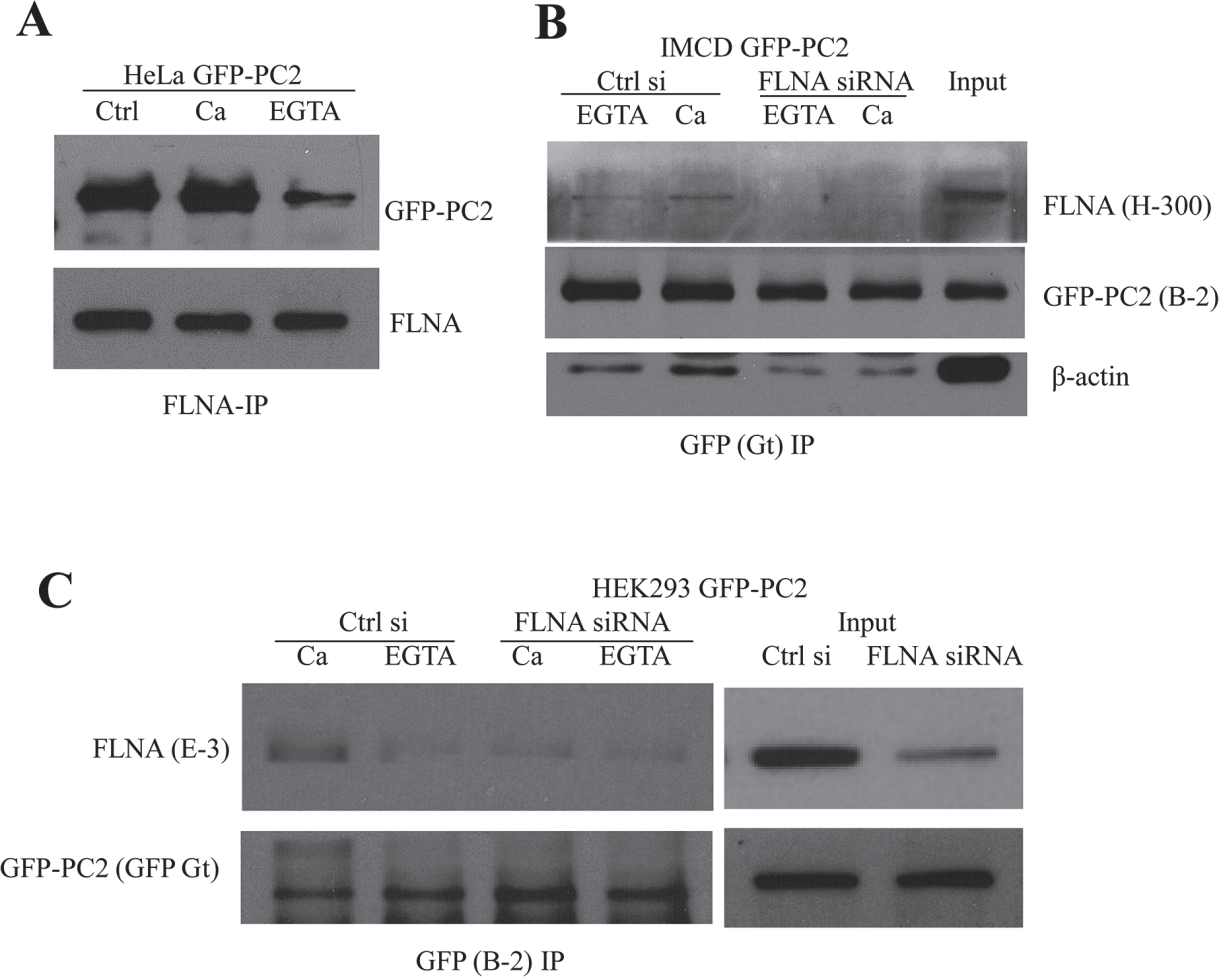
Ca dependence of the physical interaction between PC2 and FLNA. A, co-IP experiments showing Ca-dependent binding between FLNA and PC2 in HeLa cells. HeLa cell lysate with GFP-PC2 transient over-expression was equally split into three samples and added with none (Ctrl), 1 mM Ca or 1 mM EGTA (all final concentrations). FLNA (E-3) and GFP (EU4) antibodies were used for IP and IB detection, respectively. B and C, co-IP experiments showing the effect of Ca on the FLNA-PC2 binding in GFP-PC2 stably expressing IMCD (B) and HEK293 (C) cells with and without FLNA KD.

While our current study showed a stabilizer effect of FLNA on PC2 protein expression, we previously reported that FLNA inhibits PC2 cation channel activity that shows high permeability to Ca [21]. We reasoned that their physical binding should be for the purpose of (channel) functional regulation. Thus, it is possible that the FLNA-PC2 binding is to regulate the Ca entry through PC2. If this is the case, their binding may be Ca-dependent. For this we performed co-IP assays between FLNA and PC2 using HeLa cells and added a final concentration of 1 mM EGTA (to chelate Ca), 1 mM Ca, or none to the lysates. Indeed, we found that Ca chelation by EGTA substantially decreases the FLNA-PC2 binding (Fig 7A), indicating a strong Ca-dependent binding between the two proteins. No significant effect was observed for addition of 1 mM Ca, presumably because the Ca concentration in the cell lysate is already saturated for the binding between PC2 and FLNA that normally should face only sub-micromolar cytoplasmic Ca in living cells. Similar Ca dependence of the PC2-FLNA binding was observed in HEK293 and IMCD cells, but not in the presence of FLNA KD by siRNA (Fig 7B and 7C). This Ca-dependent PC2-FLNA interaction suggests a dynamic regulation of Ca flux through PC2 by FLNA that increased intracellular Ca concentration promotes the binding of FLNA to PC2 to reduce Ca leak from the ER thereby preventing further intracellular Ca increase.

## Discussion

Because too much or too little of PC2 is pathogenic [14,15], understanding how PC2 is regulated by cellular factors is of great importance. Recently we reported protein-protein interaction between filamin and polycystin-2 (PC2), through which filamin inhibited PC2 channel function [21]. In the current study we have demonstrated how FLNA regulates the stability and surface membrane expression of PC2 protein in human cultured cells, and how FLNA interacts with PC2 in a Ca-dependent manner. In particular, we have found that FLNA stabilizes PC2 on the cell surface by forming a protein triplex PC2-filamin-actin, and the physical interaction of PC2-FLNA are Ca-dependent.

Filamins are well known structural proteins that act as actin organizers, membrane stabilizers and scaffolds [35]. It was reported that FLNA stabilizes the surface membrane localization and/or inhibit degradation of several target proteins such as the Ca-sensing receptor, calcitonin receptor, chloride channel CFTR, ENaC, dopamine receptor, inwardly rectifying potassium channel Kir2.1, and pacemaker channel HCN [35–41]. For example, FLNA interacts with Ca-sensing receptors in M2 and HEK cells to increase its PM and cellular levels by preventing degradation [41]. FLNA inhibits the degradation of calcitonin receptor and is critical in maintaining its normal recycling from endosomes to the surface membrane in HEK293 cells [39]. FLNA also stabilizes the PM localization of dopamine D2 receptors and potassium channel Kir2.1 but has no effect on the total expression of the receptors [37,38]. Further, FLNA is critical for podosome stabilization for macrophage mesenchymal migration [42]. FLNC may also play important roles in protein stabilization and degradation, as mutations in FLNC can cause myofibrillar myopathy characterized by disintegration of myofibrils, massive formation of protein aggregates, and altered degradation pathways within skeletal muscle fibers [43]. Thus, our current study allows adding PC2 as a novel regulation target of FLNA, in terms of PM localization and protein degradation. On the other hand, in view of similar effects of FLNA on PC2 and other membrane proteins listed above, our finding that protein complex PC2-FLNA-actin is critical for PC2 stability and prevention of degradation suggests that the “membrane protein-FLNA-actin” link may be a general way by which many membrane proteins are anchored to actin filaments and stabilized.

It has been reported that filamin links several membrane proteins to the actin cytoskeleton, such as dopamine receptors, platelet glycoprotein Ibα, β-integrin and FcγRI [37,44–46], but there has never been direct and firm demonstration as to how filamin mediates the link. In fact, most evidence came from co-IP assays showing interactions between pairs of two proteins among membrane protein, filamin and actin. However, showing interactions between all three possible pairs does not guarantee the existence of a membrane protein-filamin-actin triplex. Because of this, we carried out co-IP assays between PC2 and actin by altering the level of FLNA in A7 and HeLa cells via FLNA shRNA, and found that FLNA is critical in the complexing between PC2 and actin (Fig 6A and 6B), which demonstrated the presence of a protein triplex in the form of PC2-FLNA-actin. This was further supported by increased recruitment of actin molecules to the surface membrane when FLNA is present and is not interfered by blocking peptide FLNAC (Fig 6D).

Several studies indicated that the protein level of PC2 is critical for cell function and needs to be strictly regulated within a narrow range. Immunohistochemistry studies using staged mouse embryos indicated that PC2 expression is developmentally regulated and that this regulation is important for embryo development [47]. In adult mice, PC2 was also reported to be markedly up-regulated by renal ischaemic injury [48–50], indicating the importance of PC2 for the recovery from ischaemia. Studies of PC2-dependent ADPKD in mouse model also showed that both PC2 knock-out and knock-in mice develop typical renal cysts and an increase in cell proliferation and apoptosis, which are reflective of human ADPKD phenotypes [14,15]. However, mechanisms of how PC2 protein level is regulated are still not well understood. Generally, the steady-state protein level is defined by both synthesis and degradation. Although some transcription factors such as E2F, EGRF and SP1 were predicted by computational analyses to bind with the PKD2 promoter, little experimental data on PKD2 transcriptional regulation have been published [51]. On the translation level, it was reported that microRNA miR-17 down-regulates PC2 by binding with its 3’ untranslated region [52] and that this binding site is also recognized by the RNA-binding cystic protein bicaudal C, which antagonizes the repressive activity of miR-17 [53]. Previously, we reported that PC2 is regulated by endoplasmic reticulum associated degradation (ERAD) through the ubiquitin-proteasome system by interacting with Herp, an ubiquitin-like protein implicated in regulation of ERAD [31]. Our current study revealed a novel mechanism by which a membrane protein can be stabilized, namely through forming protein complex PC2-FLNA-actin for anchorage to the actin filaments.

We also examined whether FLNA affects the mRNA level of PC2 in HeLa, HEK293, A7 and M2 cells by reverse transcript (RT)-PCR and real-time RT-PCR, and found that the PKD2 mRNA level is much lower in the presence of FLNA, eg, the mRNA level in A7 cells represented only 41 ± 5% (N = 5; p < 0.001) of that in M2 cells, which roughly accounted for the observed lower synthesis (Fig 1C). The mechanism of how FLNA regulates the mRNA level of PKD2 remained to be determined by future studies. However, it was previously reported that cytoplasmic FLNA through binding with transcription factors such as PEBP2/CBF and p73α regulates the transcription of several genes including interlukin-3, T cell receptors, and cell cycle inhibitor p21 Waf1/Cip1 [54–56]. Therefore, it is possible that PKD2 gene is a downstream target of a FLNA-regulated transcription factor.

In summary, our present study found that FLNA is an important regulator of PC2 that it prevents PC2 degradation and stabilizes surface membrane PC2 through forming protein triplex PC2-FLNA-actin for anchoring to the cytoskeleton. This mechanism of stabilization may be important for cystogenesis of ADPKD. However, ADPKD renal cysts arise from different nephron segments (proximal and distal tubules) with different characteristics [57] which can't be modeled by cultured cell lines. This should be considered when interpreting the data obtained in the present study. Our finding of PC2 stabilized by filamin and actin may also be applicable to other membrane proteins of which the stability and degradation are similarly regulated by filamin. On the other hand, FLNA binds PC2 and inhibits its channel function through direct binding [21], and the PC2-FLNA binding was found to be Ca-dependent (Fig 7). With regard to what is the net effect of FLNA, we think that when the cytoplasmic Ca concentration is high, FLNA through direct binding shuts down PC2-mediated Ca entry (from the extracellular space or ER) to avoid Ca overloading; Conversely, when Ca is low, FLNA dissociates from PC2 thereby removing the inhibition to allow Ca entry via PC2. Because the intracellular Ca concentration would frequently change in time and space filamins may still be able to maintain PC2 stability on the membrane while accomplishing its Ca-dependent binding with and functional regulation of PC2.
